# RNA-Seq reveals new DELLA targets and regulation in transgenic GA-insensitive grapevines

**DOI:** 10.1186/s12870-019-1675-4

**Published:** 2019-02-18

**Authors:** Jie Arro, Yingzhen Yang, Guo-Qing Song, Gan-Yuan Zhong

**Affiliations:** 1 USDA-ARS Grape Genetics Research Unit, Geneva, NY 14456 USA; 2 Department of Horticulture, Michigan State University, East Lansing, MI 48823 USA

**Keywords:** CONSTANS, DELLA, Flowering, Grapevine, HOMEODOMAIN, Perennial, TFL1, Transgenic GA-insensitive

## Abstract

**Background:**

Gibberellins (GAs) and their regulator DELLA are involved in many aspects of plant growth and development and most of our current knowledge in the DELLA-facilitated GA signaling was obtained from the studies of annual species. To understand GA-DELLA signaling in perennial species, we created ten GA-insensitive transgenic grapevines carrying a DELLA mutant allele (*Vvgai1*) in the background of *Vitis vinifera* ‘Thompson Seedless’ and conducted comprehensive analysis of their RNA expression profiles in the shoot, leaf and root tissues.

**Results:**

The transgenic lines showed varying degrees of dwarf stature and other typical DELLA mutant phenotypes tightly correlated with the levels of *Vvgai1* expression. A large number of differentially expressed genes (DEGs) were identified in the shoot, leaf and root tissues of the transgenic lines and these DEGs were involved in diverse biological processes; many of the DEGs showed strong tissue specificity and about 30% them carried a DELLA motif. We further discovered unexpected expression patterns of several key flowering induction genes *VvCO, VvCOL1* and *VvTFL1*.

**Conclusions:**

Our results not only confirmed many previous DELLA study findings in annual species, but also revealed new DELLA targets and responses in grapevine, including the roles of homeodomain transcription factors as potential co-regulators with DELLA in controlling the development of grapevine which uniquely possess both vegetative and reproductive meristems at the same time. The contrasting responses of some key flowering induction pathway genes provides new insights into the divergence of GA-DELLA regulations between annual and perennial species in GA-DELLA signaling.

**Electronic supplementary material:**

The online version of this article (10.1186/s12870-019-1675-4) contains supplementary material, which is available to authorized users.

## Background

Gibberellins arguably has the widest role to play in the whole life cycle of a plant. Plants tightly regulate the availability of GA by coordinating the activation and deactivation of multiple biosynthesis genes of GA in different tissues at various developmental stages [1]. The flow rate and availability level of bioactive GA in various tissues are regulated by a de-repressible system in which GA interacts with its receptor *GA INSENSITIVE DWARF1* (GID1) and negative regulator, DELLA, to form a trimeric GA:GID1:DELLA complex [2]. As a key component in the GA signaling cascade, DELLA genes, such as *Rht1* from wheat and *GIBBERELLIN INSENSITIVE* (*GAI*) from *Arabidopsis*, have been extensively studied [3]. These genes all carry a conserved DELLA amino-acid domain in their protein sequences. A critical gain-of-function mutation in the DELLA domain prevents the formation of a stable trimeric complex with GA:GID1 required for its eventual degradation, and thus instead maintains the negative regulation of GA signaling [4]. As a result, GA-deficient plants show many abnormal developmental characteristics such as smaller darker leaves and reduced internode length [5]. In addition to basic characterization of DELLA protein genes in the GA signal transduction [6], many DELLA target genes which control development of various traits in model and annual species, have also been identified [7]. In contrast, progress in understanding DELLA regulation in perennial woody plants lags considerably behind, although some DELLA knowledge has been obtained from transgenic poplar research; metabolic and phenotypic changes in transgenic GA-deficient poplar [8] were found similar to their  *Arabidopsis* mutant counterpart [9]. Similar to *Arabidopsis gai* mutants, transgenic poplar carrying non-degradable DELLA gene (i.e. *gai,* a mutant version of *GAI*) were dwarf but insensitive to GA-mediated restoration to wildtype [10, 11].

Grapevine (*Vitis*) is a perennial woody species with significant economic value. Impact of DELLA-mediated GA on vine growth and development was first found in a natural DELLA mutant derived from a somatic variation in the L1 meristem layer of the grape cultivar *V. vinifera* ‘Pinot Meunier’ [12]. The mutant vine carries a copy each of the wildtype *GIBBERELLIC ACID-INSENSITIVE1* (*VvGAI1*) and mutant (*Vvgai1*) and manifests reduced internode length and short plant stature, which is a hallmark trait of DELLA mutants in many species. The mutant vine also shows continuous flowering and bears inflorescences at most nodes after the first inflorescence appears. Apparently, both GA and DELLA proteins are involved in grape’s inflorescence formation, flower induction and berry development [13].

To elucidate DELLA-mediated roles in grapevine development, we created transgenic lines in the background of a table grape cultivar, *V. vinifera* ‘Thompson Seedless’, carrying a grape mutant DELLA gene, noted as *Vvgai1* [14]. We analyzed the RNA-Seq profiles of shoot, leaf and root tissues of non-transgenic and representative transgenic lines and affirmed in grapevine the presence of a DELLA-centered feedback mechanism that maintains the GA homeostasis [15] and also the intricate interactions of DELLAs with numerous transcription factors controlling plant development and growth as were found in annual species [16]. We further discovered DELLA’s possible roles in the induction of the anlagen, the distinct vegetative meristem for tendril/inflorescence development in grapevine [17], through coordination with meristem regulators. Moreover, we discovered unexpected expression patterns of several key flowering induction genes, including grapevine *CONSTANS* (*VvCO), CONSTANS 1* (*VvCO1),* and *TERMINAL FLOWER 1 (VvTFL1 or CENTRORADIALIS)*, which were in sharp contrast with what were found in annual species. These findings provide insights into how DELLA genes regulate grapevine development and growth, especially in relation to flowering, and fill some critical knowledge gaps in this important research area between annual and perennial species.

## Results

### Transgenic *Vvgai1* expression and phenotypes

**Fig. 1:**
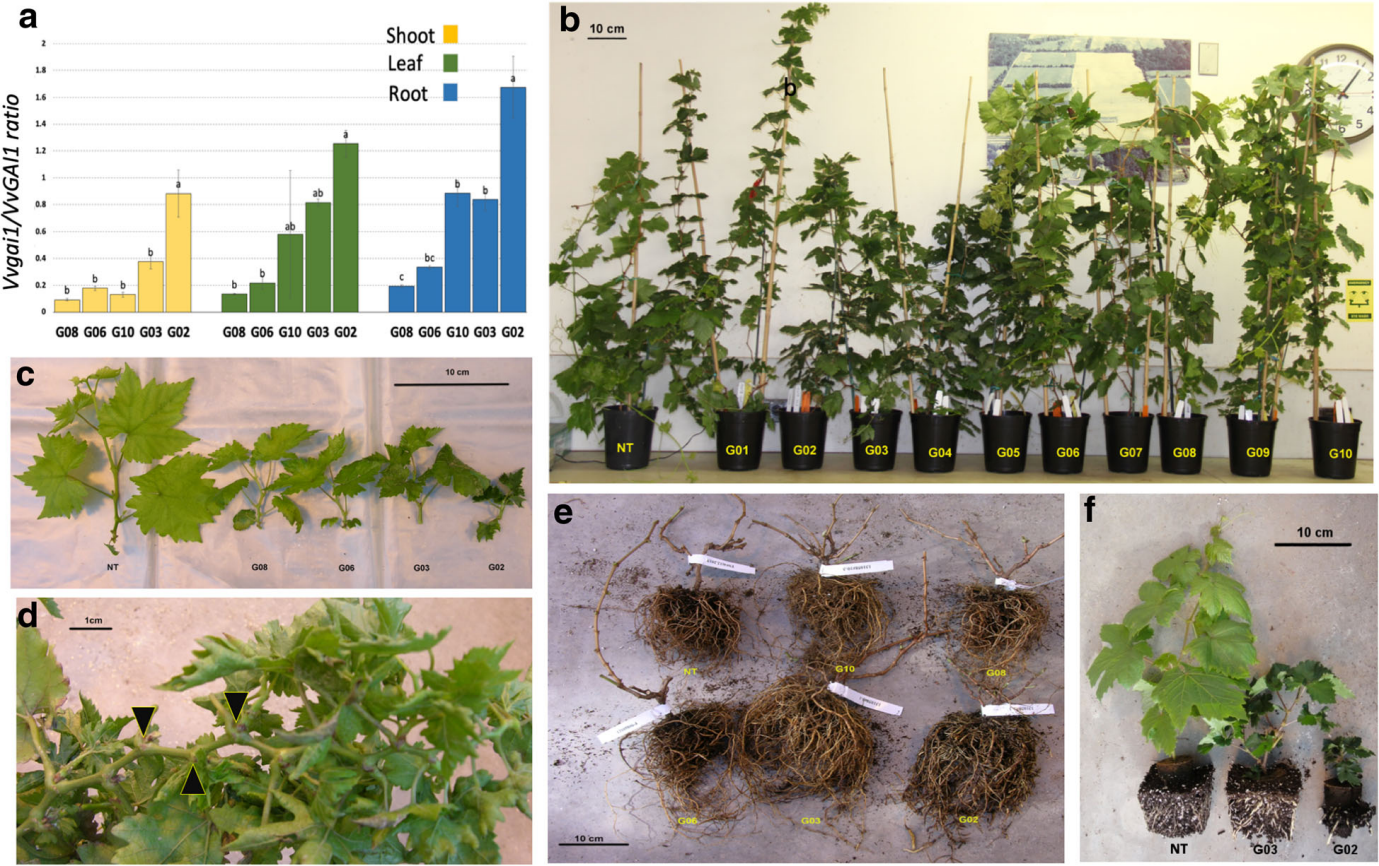
*Vvgai1* expression levels and phenotypes of non-transgenic control (NT) and transgenic lines (denoted with prefix G). **a** qRT-PCR of *Vvgai1* expression in different tissues (bar plot indicated by different letters are significantly different at pval≤0.01); **b** seven-month-old transgenic vines with varying plant height; **c** shoot and leaf characteristics (e.g. leaf size, color and curling); **d** Arrows indicate an example of abnormal tendril distribution pattern (three consecutive nodes without a tendril) in a transgenic line; **e** seven-month-old transgenic vines with varying root mass; **f** three-month-old NT, G03 and G02 vines

Ten *Vvgai1* transgenic vines were generated in this study and five representative ones covering a range of mutant phenotypic variations were chosen for RNA-Seq analysis. The internode length of four transgenic lines were significantly shorter than the NT in various degrees, with approximate three-fold reduction for the most severe transgenic line G02 (Fig. 1a and b, Table 1). More severe dwarf lines had smaller shoots and leaves that were darker and curled at the edges (Fig. 1c). The distinctive *V. vinifera* shoot patterning of two consecutive nodes with a tendril followed by node without a tendril [18] was proportionally disrupted with a higher frequency of tendril skips among the shorter dwarf lines. The tendrils also appeared progressively late among the severe dwarf vines, as late as the tenth node in G03 compared to the fifth in the NT (Fig. 1d, Table 1). No tendrils were observed for the most severe G02 line even after 1 year growth in greenhouse conditions (data not shown). More severe dwarf lines had proportionally enlarged and denser roots (Fig. 1e, f) and showed poorer root establishment.

**Table 1:** Phenotypic and *Vvgai1* expression variation observed in NT and five transgenic lines (denoted with prefix G) in the study

Genotype ID	Leaf weight (g)^4^	Internode length (cm, average of 10 nodes)^4^	Average no. of lateral branches with 5 or more nodes^4^	Average node position having first tendril^4^	Average no. of tendril per node^4^	Chlorophyll content ^2^	*Vvgai1* in shoot^3^	*Vvgai1* in leaf^3^	*Vvgai1* in root^3^
G02	0.31*	1.18**	0.00	NA^1^	NA^1^	1.58**	0.89^a^	1.25^a^	1.67^a^
G03	0.90	1.87**	0.33*	10.43**	0.54**	1.36*	0.37^b^	0.81^ab^	0.84^b^
G10	0.71*	2.41**	2.33	5.47	0.66	1.04	0.13^b^	0.59^ab^	0.88^b^
G06	1.20	2.71*	0.60**	7.54**	0.62**	1.16*	0.18^b^	0.22^b^	0.33^c^
G08	0.86*	3.36	2.00	7.20*	0.65	0.95	0.09^b^	0.13^b^	0.19^c^
NT	1.06	3.67	5.50	5.86	0.68	1.00	–	–	–

*, ** Significantly different from NT at *p* < 0.05 and < 0.01, respectively

^1^No observable tendrils at the time of sampling about 5 months after potting

^2^A ratio of observed absorbance reading of a transgenic line to the NT’s on the basis of a bulked sample of five mature leaves

^3^A ratio of *Vvgai1/VvGAI1* expression derived from qRT-PCR; means followed by the same letters were not significantly different at *p* < 0.01

^4^Measurements were taken from five biological replicates

As expected, *Vvgai1* expression was found in all the five transgenic lines but not in the NT using digital qRT-PCR. Among the five transgenic lines, the most severe dwarf G02 showed statistically the highest *Vvgai1* expression in all three tissues (Table 1, Fig. 1a). These results suggest that there was a positive correspondence between the relative *Vvgai1* expression levels (*Vvgai1/VvGAI1* ratios) and the observed severity of phenotypic changes among the transgenic lines.

### Differentially expressed genes associated with transgenic *Vvgai1* expression

**Fig. 2:**
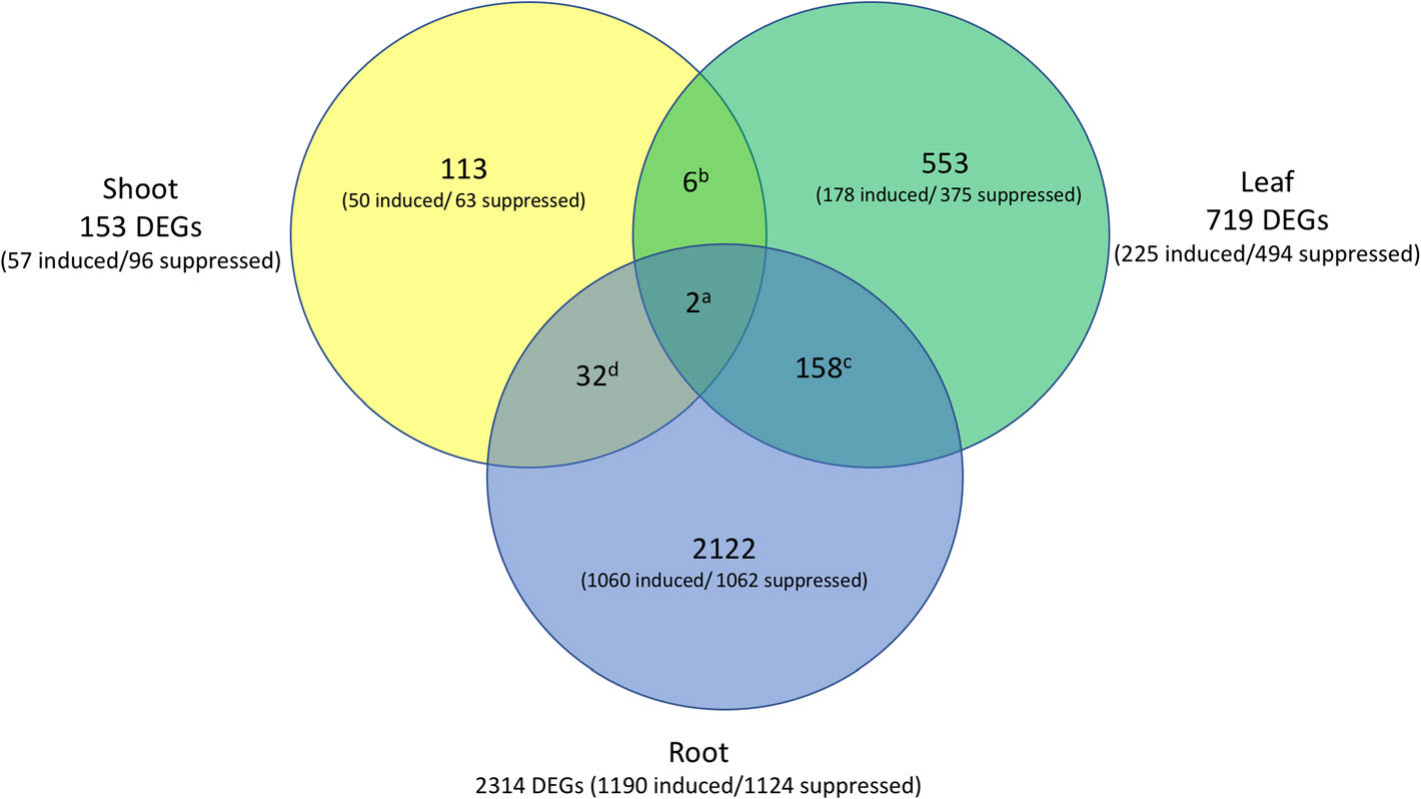
Distribution of the 2986 DEGs (1.5x-fold changes at FDR ≤ 0.01) among the shoot, leaf and root tissues. a:2 suppressed in all tissues; b:4 suppressed in both shoot and leaf, 1 induced in both shoot and leaf, 1 induced in shoot, but suppressed in leaf; c:8 suppressed in both leaf and root, 13 induced in both leaf and root, 33 induced in leaf, but suppressed in root, 104 suppressed in leaf, but induced in root; d:17 suppressed in both shoot and root, 4 induced in both shoot and root, 2 induced in shoot, but suppressed in root, 9 suppressed in shoot, but induced in root

Differential gene expression analyses in the leaf, shoot and root tissues were separately conducted for the five transgenic lines. More severe dwarf lines generally have more DEGs, although such trend was not observed in the root tissue (Fig. 2, Additional file 1: Table S1). To capture maximum numbers of DEGs associated with *Vvgai1* with the least false positives, we opted to retain only those DEGs (1.5x-fold changes at FDR ≤ 0.01) in the most severe transgenic line G02 and concurred in at least one of the four remaining transgenic lines. We also identified 25 genes originally filtered out for not having the minimum reads (i.e. 1CPM) in all the 12 libraries, but were in fact expressed only in the transgenic lines and suppressed in the NT, or vice-versa (Additional file 2: Table S2). This recovered set had several cell-wall related genes induced only in the leaf of the transgenic lines and a key flowering gene *SHORT VEGETATIVE PHASE* (*VvSVP)* highly suppressed in the root of the transgenic lines. The final numbers of DEGs for subsequent analyses were 153, 719, and 2314 for shoot, leaf and root, respectively, totaling to 2986 genes significantly differentially expressed at least once in the three sampled tissues (Fig. 2).

We validated the RNA-Seq data by analyzing the digital RT-PCR expression of 30 notable genes related to DELLA regulation from the same tissues. The RT-PCR and RNA-Seq datasets were in a good agreement with a general correlation coefficients (r) ranging from 0.77 to 1.0 (Additional file 3: Table S3).

### Gene ontology (GO) analysis

The annotated DEGs in the shoot and leaf tissues were comprised of about twice as many suppressed (96 for the shoot and 494 for the leaf) as induced ones (57 for the shoot and 225 for the leaf), while the root had about the same (1124 suppressed and 1190 induced) (Fig. 2). The enriched GO terms have large overlaps of the member genes, and a semantic clustering analysis revealed about ten common interdependent biological themes (Table 2). This is not a surprise as the arbitrary process of clustering involved a considerable number of master growth regulators with broad biological roles known to coordinate plant’s morphogenetic changes and responses to stimuli, such as the transcription factors *NAM/ATAF1,2/CUC2* (*NAC)*, *WRKY*, ﻿*SQUAMOSA PROMOTER BINDING PROTEIN LIKE* (*SPL)*, *TB1-CYC-PCF* (*TCP)* and *﻿THREE-AMINO-ACID-LOOP-EXTENSION (TALE)* class of homeoproteins. There were also numerous biosynthesis and signaling genes belonging to hormones auxin, cytokinin, brassinosteroids, and ethylene.

**Table 2:** Enriched GO terms and their average correlations (Pearson r) with *Vvgai1* expression levels in the shoot, leaf and root transcriptome

Tissue	Class	Representative	GO annotation	GO ID	Enrichment FDR (log10)^b^	Ave r pval (log10)^c^	Notable genes^d^
		GO cluster term ^a^					
Shoot	Negatively correlated	1	amino sugar catabolic process	GO:0046348	3.4	5.77	MAPK3, MATE EFFLUX, ATCHITIV, ATCHIA
		1	cell wall macromolecule catabolic process	GO:0016998	3.66	5.65	
		1	hormone catabolic process	GO:0042447	3.01	3.41	
		2	cellular process	GO:0009987	4.22	4.58	
		3	response to red light	GO:0010114	3.6	5.47	PR4, YSL3, GA2ox1, PRR7, BES1, LAC1, GRF5, DWARF4, TEMPRANILLO 2
		3	response to chemical	GO:0042221	3.05	4.63	
		3	response to abiotic stimulus	GO:0009628	4.46	4.58	
		4	developmental process	GO:0032502	3.7	4.85	
		7	floral meristem determinacy	GO:0010582	3.01	5.64	SPLs, VvSVP, ACO4, CKX, CKX5, PRX66
		7	anatomical structure development	GO:0048856	3.49	5.11	
Leaf	Positively correlated	1	phenylpropanoid metabolic process	GO:0009698	12.48	19.72	FLS1, ATCHS, 2-ODD, UGT86A2
		1	aromatic compound biosynthetic process	GO:0019438	3.8	12.46	
		10	oxidation-reduction process	GO:0055114	6.01	15.09	CCD1, MES10,CAT2
		10	secondary metabolic process	GO:0019748	6.07	13.8	
	Negatively correlated	1	DNA replication initiation	GO:0006270	4.7	4.05	CDC45, MCM2, GRFs, MYB59, NAC71, WRKY 57
		1	polysaccharide metabolic process	GO:0005976	2.86	9.22	
		10	lipid metabolic process	GO:0006629	2.74	9.77	
		7	regionalization	GO:0003002	4.68	10.59	SHR, YABBY1, AS1, CIB4, DWARF4
		7	shoot system morphogenesis	GO:0010016	3.19	9.57	
		7	pattern specification process	GO:0007389	5.49	9.56	
		8	chromatin organization	GO:0006325	2.63	4.81	CDC45, MCM2
Root	Positively correlated	1	toxin catabolic process	GO:0009407	3.52	6.66	SCR 21, VvDELLA1, VvDELLA2, PIF1, KNAT3, ARF16, ARF11, VvTFL1
		1	phenylpropanoid metabolic process	GO:0009698	12.48	5.07	
			cellular biosynthetic process	GO:0044249	3.8	4.84	
		1	hydrogen peroxide catabolic process	GO:0042744	4.42	4.38	
		3	ethylene-activated signaling pathway	GO:0009873	3.96	6.9	KO1, GA3ox, GA20ox1, GASA6, LRR family, UMAMIT42
		3	response to chemical	GO:0042221	19.33	4.93	
		3	response to stress	GO:0006950	23.53	4.74	
		3	response to abiotic stimulus	GO:0009628	4.01	4.36	
		7	coenzyme A metabolic process	GO:0015936	4.34	6.83	PIF1, VvTFL1, C 4, MAP65–6, TUB8, SHR,CYP722A1, FLA4, MFS family,
		7	cell recognition	GO:0008037	3.31	5.15	
		7	indole-containing compound metabolic process	GO:0042430	3.07	4.69	
		7	organic acid metabolic process	GO:0006082	10.66	4.44	
	Negatively correlated	1	gene expression	GO:0010467	3.37	4.88	CHS, LACASSE, 2-ODD, AP2/ERF, WRKY
		1	cellular macromolecule biosynthetic process	GO:0034645	4.29	4.78	
		1	cellular biosynthetic process	GO:0044249	3.33	4.64	
		3	gibberellic acid mediated signaling pathway	GO:0009740	6.55	8.79	PIN3, EIN3, ARR5, ARR9, OMR1, ACS1
		3	ethylene-activated signaling pathway	GO:0009873	3.55	6.72	
		3	response to red light	GO:0010114	5.24	6.31	
		3	response to chemical	GO:0042221	4.07	5.18	
		3	response to abiotic stimulus	GO:0009628	9.32	4.83	
		7	anatomical structure development	GO:0048856	4.39	4.46	
		4	developmental process	GO:0032502	3.96	4.4	
		5	localization	GO:0051179	4.49	3.87	
		6	metabolic process	GO:0008152	9.78	4.19	
		9	response to stimulus	GO:0050896	6.39	4.61	

^a^GO cluster term: 1:Aromatic compound catabolism; 2:Cellular process; 3:Detoxification of nitrogen compound; 4;Developmental process; 5:Localization; 6:Metabolism; 7:Pattern specification process; 8:Protein polymerization; 9:Response to stimulus; and 10:Secondary metabolism

^b^Enrichment pval (log_10_ scale) as determined by PlantMet GenMap

^c^Average expression correlation (Pearson’s r pval (log_10_ scale)) of the member genes with the expression level of *Vvgail* in the enriched GO term

^d^While these genes were listed under a specific GO cluster, many of them were present in other clusters as well

### Expression profiles of key GA signaling genes

**Fig. 3:**
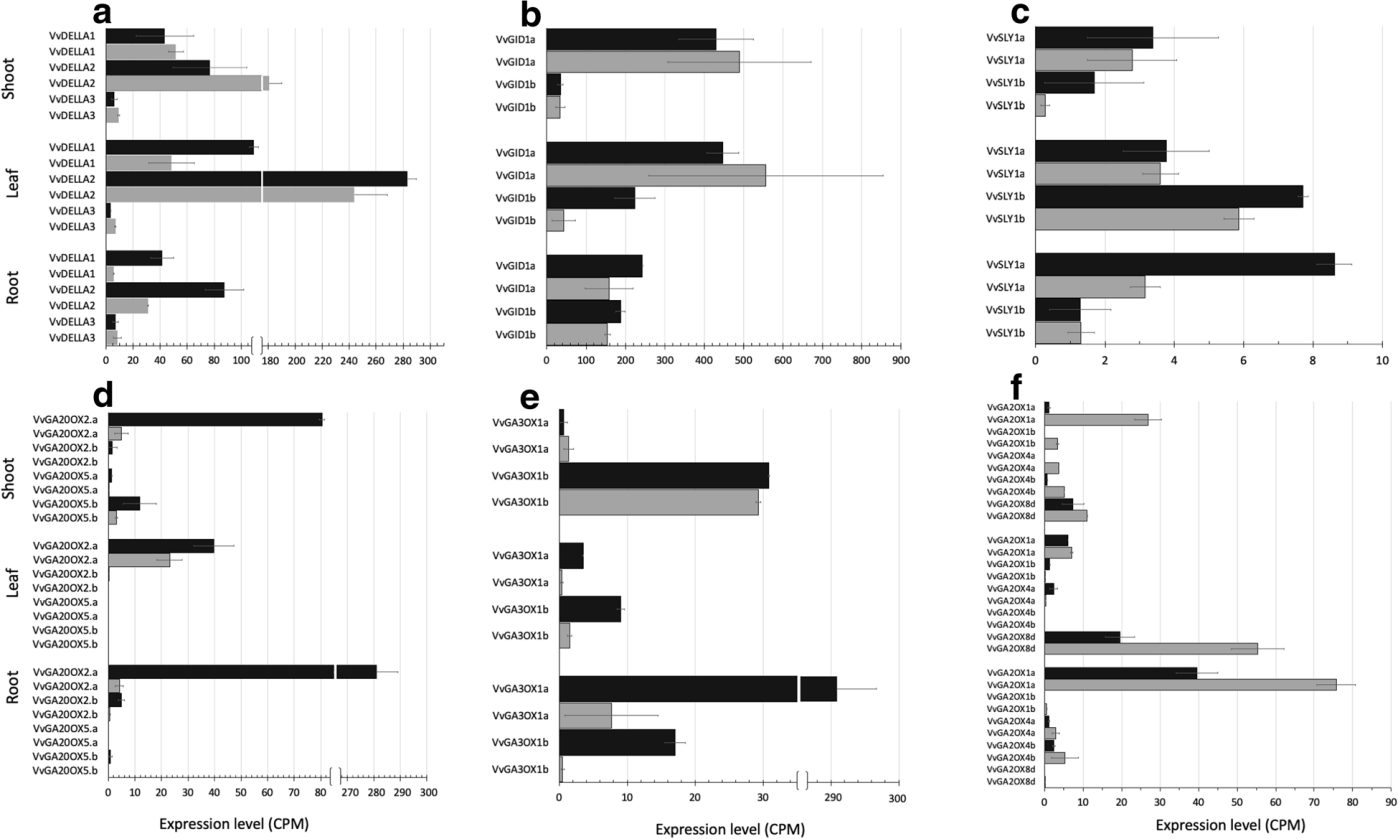
Expression profiles of GA signaling genes in NT and the most severe transgenic line G02 in the shoot, leaf and root tissues. Black bar = G02; Grey bar = NT. **a** *VvDELLAs* (note: *VvDELLA1* is synonymous to *VvGAI1;* expression levels were corrected on the basis of the relative *Vvgail* and *VvGAI1* ratios derived from the qRT-PCR results), **b** *VvGID1*, **c** *VvSLY1*, **d** *VvGA20ox*, **e** *VvGA3ox*, and **f** *VvGA2ox*

Similar to the currently accepted model in *Arabidopsis* [6], the grapevine GA signaling are comprised of three DELLA homologues of *VvDELLA1*, *2* and *3*, two GA receptor GID1 of *VvGID1a* and *VvGID1b*. Grapevine, however, have two proposed F-box SLY1 genes, *VvSLY1a* and *VvSLY1b* [19]. *VvDELLA1* (synonymous to *VvGAI1*) in the NT was more abundant in the shoot and leaf (~ 50 CPM), about five folds higher than that in the root (~ 10 CPM). *VvDELLA1* in the transgenic G02 line, however, was differentially expressed with about two folds higher than the NT in the leaf and root, but not in the shoot (Fig. 3a). *VvDELLA2* had the highest expression in the root among the three NT tissues (30–240 CPM) and its expression in the root, but not in the other two tissues, was increased more than twice in the transgenic G02 line. *VvDELLA3* was consistently expressed at low levels in all three tissues in both NT and transgenic background (~ 10 CPM). The expression profiles of *VvDELLA1* and *VvDELLA2* were validated by RT-PCR with high correlation coefficients (r = ~ 0.95, Additional file 3: Table S3). The endogenous *VvDELLAs* seemed responsive to and inducible by the transgenic *Vvgai1*.

The two homologues of GA receptor GID1 were abundantly expressed in all tissues in the NT. In the transgenic lines, *VvGID1b* showed five folds of expression increases in the leaf (~ 40 CPM to ~ 220 CPM) (Fig. 3b). The two grapevine F-box genes, *VvSLY1a* and *VvSLY1b,* consistently showed low levels of expression (< 10 CPM) in both NT and transgenic background (Fig. 3c).

Homologues of the GA biosynthesis genes *GA3 oxidase* (*GA3ox)* and *GA20 oxidase* (*GA20ox),* another group of recipients of the DELLA-controlled feedback mechanism (like *GID1*) [20], were induced in all the three sampled tissues but at different levels, seemingly preserving the observed tissue-specificities of this multi-gene family [21]. *VvGA20ox2a* showed 15-, 2- and a dramatic 50 folds of increases in the shoot, leaf and root, respectively (Fig. 3d). In a similar manner, *VvGA3ox1a* was induced by more than four folds in the leaf, and 10 folds in the root (Fig. 3e). In consonance, the GA deactivating *GA2ox* genes were generally suppressed in the transgenic background. The largest suppression was observed for *VvGA2ox1a,* which decreased by about nine folds (from 28 CPM to 3 CPM) and two folds (76 CPM to 40 CPM) in the shoot and root, respectively. *VvGA2ox8a* was also suppressed with a decrease of about 2.5 folds (55 CPM to 20 CPM) in the leaf (Fig. 3f). These results affirm the positive feedback mechanism facilitated by DELLA to *GID1* and the biosynthesis genes *GA20ox* and *GA3ox* [15]. Interestingly in this study, we observed that such feedback signal appeared tissue-specific, i.e. different *GID1* and GA biosynthesis homologues induced in different tissues.

### Expression profiles of some key genes influencing shoot, leaf, and root development

**Fig. 4:**
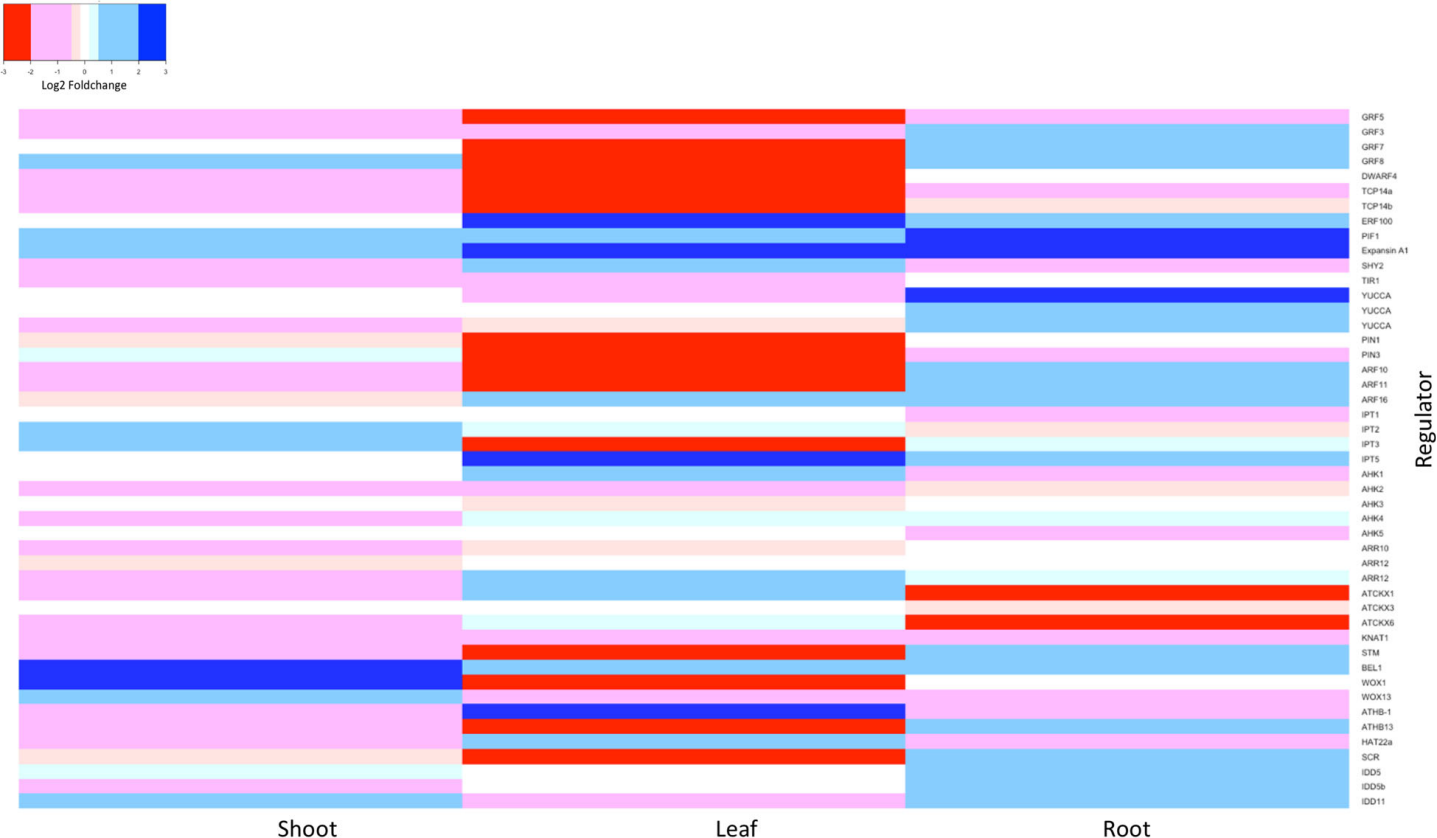
Differential expression profiles of key regulators influencing shoot, leaf, and root development in the transgenic line G02

A number of highly responsive DEGs observed are regulators of developmental processes. Among the highly suppressed were members of the *GROWTH REGULATING FACTOR* (GRF) gene family, important regulators mostly found in intercalary meristem and leaf primordia [22]. For example, the homologue *GRF5* controlling cell number related to leaf size [23] was suppressed by about 20 folds in the leaf of the G02 line (Fig. 4). The brassinosteroid biosynthesis gene *DWARF4*, another key gene synergistically associated with auxin [24] and GA regulation [25] and whose loss-of-function mutants were dwarfs with round and dark-green leaves in shortened petioles [26], was suppressed in all three tissues, but especially in the leaf of the G02 line by nine folds (Fig. 4). The TCP transcription factor, a master regulator that influences plant height, leaf curvature [27] and tendril phylloctatic patterning in grapevine [28], was suppressed at four to nine folds in the shoot and leaf of the transgenic G02 line (Fig. 4).

Genes for light-mediated developmental processes were notably induced. *ERF105*, a gene active during intense light and freezing stress conditions [29, 30], was induced by seven and three folds in the leaf and root, respectively, and the *PHYTOCHROME INTERACTING FACTOR 1 (PIF1)*, a major gene involved in chloroplast production through maintenance of cell number in the leaf [31], was increased by three folds in the shoot and leaf and about eight folds in the root (Fig. 4). The cell wall loosening gene *EXPANSIN A1* was also induced about two folds in the shoot and six folds in the leaf and root of the transgenic background (Fig. 4).

### Auxin and cytokinin

Auxin and cytokinin related genes have extensive cross-talk with GA-DELLA regulation as master regulators for many growth and development processes in plants [32]. Auxin-related genes seemed generally active in all transgenic lines. *SHORT HYPOCOTYL 2 (SHY2*), a negative regulator of auxin responses [33], was negatively correlated to *Vvgai1* expression in the shoot and root with a suppressed response of about two and four folds, respectively, in the transgenic line G02 (Fig. 4). Conversely, the grape homologous gene of *TRANSPORT INHIBITOR RESPONSE 1* (*TIR1*), a positive regulator of auxin [34], was only slightly induced in the shoot but increased by about two folds in the root*.* In conjunction, two homologues of the auxin biosynthesis gene *YUCCA* showed induced responses more prominently in the root. On the other hand, related auxin transporters and response factors exhibited various responses in the sampled tissues, reflecting the dynamic auxin flux. The grape homologue of *PIN-FORMED3 (PIN3)* was suppressed and significantly negatively correlated with *Vvgai1* in their expression in the root, which was validated by our qRT-PCR (Additional file 3: Table S3). The grape homologue of *AUXIN RESPONSE FACTOR11* (*ARF11)*, one of the response factors antagonized by cytokinin when over-expressed in the root [35] was strongly induced. Interestingly, activities of cytokinin-related genes seemed unusually low in our transgenic lines. The biosynthesis genes homologous to *ISOPENTENYLTRANSFERASE2* (*IPT2*) and *IPT3* were of low expression levels (10 CPM) in the three sampled tissues. The grape *CYTOKININ OXIDASE* (*CKX)* gene family that irreversibly deactivate cytokinins [36] was suppressed in the root, although some were interestingly induced in the leaf (Fig. 4).

### Homeodomain gene family

The expressions of many homeodomain transcription factors were generally suppressed and a large number of them belonged to the family classes of *TALE*, *WUSCHEL RELATED HOMEOBOX* (*WOX*) and *HOMEODOMAIN-LEUCINE ZIPPER* (*HD-ZIP*) (Additional file 1: Table S1). These homeodomain transcription factors are extensively involved in regulation of meristem maintenance and plant form [37], and manifest disruption of shoot phyllotaxy [38]. *TALE* has two subfamilies, *KNOTTED1-like* (*KNOX*) and *BELLRINGER* (*BEL*). In the *KNOX* subfamily, *KNOTTED-LIKE FROM Arabidopsis thaliana1* (*KNAT1*) and *SHOOT MERISTEMLESS* (*STM*) are a homologous pair that synergistically interact with cytokinin in the shoot apical meristem (SAM) [39]. We observed that *STM* was suppressed in the shoot and leaf of the transgenic line G02; *KNAT1* was suppressed only in the shoot (Fig. 4); and *KNAT1* was not expressed in the leaf which is in agreement with its negative role in leaf primordia initiation [40]. *BEL1* (*BELLRINGER 1*), a member of the BEL subfamily which forms critical KNOX-BEL heterodimers for proper initiation and maintenance of the apical meristem [41], was induced in all three tissues in the transgenic line G02. WOX family genes are specialized transcription factors found during embryogenesis and lateral organ formation [42]. In transgenic line G02, *WOX1* gene was induced in the shoot but suppressed in the leaf, while the *WOX13* was suppressed in the root (Fig. 4). Furthermore, we found that the HD-ZIP family member genes were generally suppressed in the shoot and root tissues. HD-ZIP member genes are associated with organ and vascular development [43].

### Flower-induction pathways

**Fig. 5:**
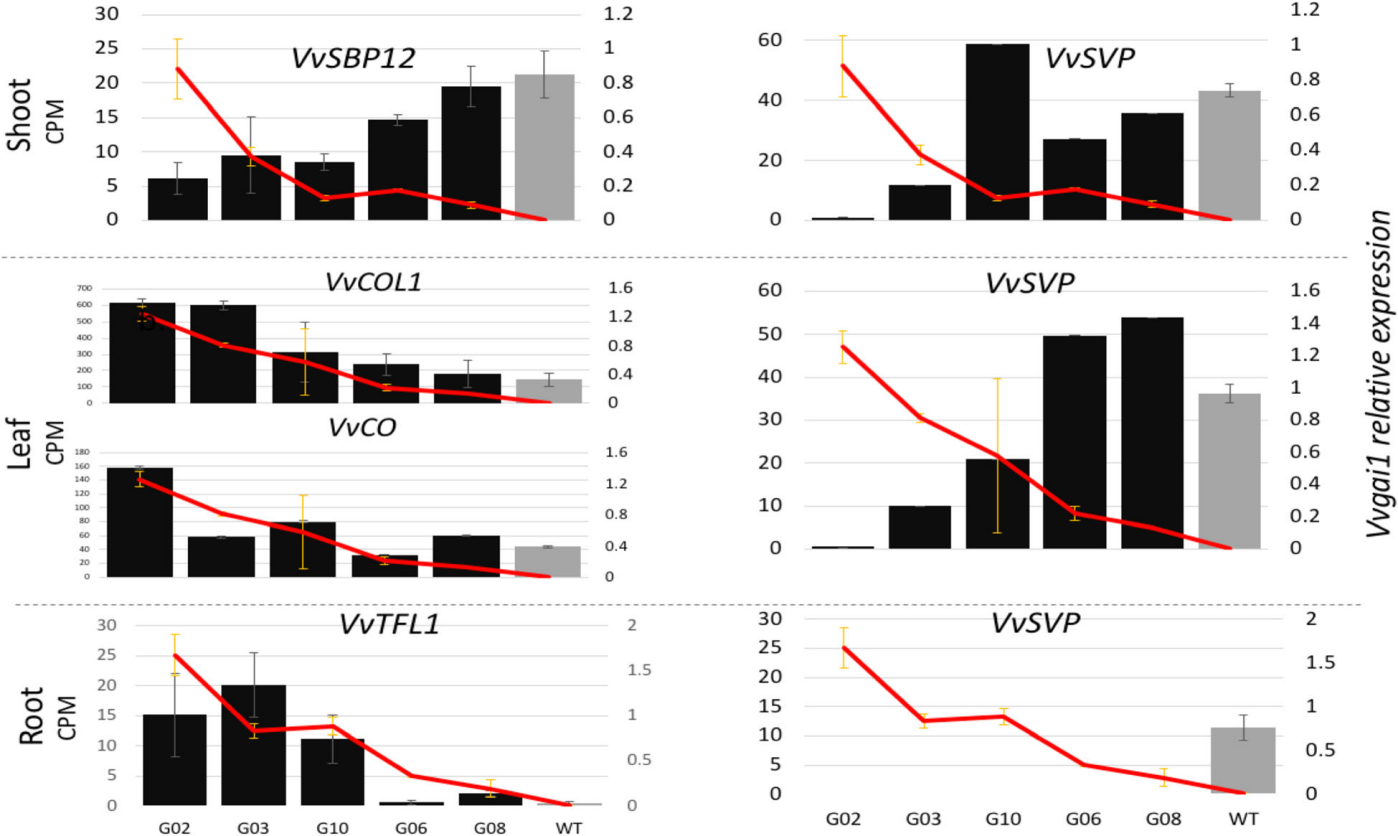
Average expression levels (CPM) of key flowering-related genes in the shoot, leaf and root tissues of five transgenic lines (black bars) arranged according to their dwarf severity and NT (grey bar); *Vvgai1* expression is presented as a red line plot (see Table 1)

Four flowering-related genes showed significant responses to *Vvgail* expression in this study. *VvSBP12* is a member of the miR156-targeted grapevine SPL genes [44] and its homologue in *Arabidopsis*, *AtSPL3*, was shown to be a target of suppression by DELLA and a target of miRNA156/− 172 phase-change regulation [45]. The *VvSBP12* was suppressed in all three tissues of the G02 line, especially in the shoot (Fig. 5). Interestingly, one of the homologues of a novel gene family associated with miRNA metabolism, HEN1 [46], showed variable response patterns among the three tissues, being induced in the shoot and leaf but strongly suppressed in the root (Additional file 1: Table S1). On the other hand, the two *CONSTANS* homologues in grapevine, *VvCOL1* and *VvCO*, were induced in all three tissues (Fig. 5), which is in contrast in *Arabidopsis’* where they were reported as targets of suppression by DELLA [47]. Interestingly, *VvTFL1* gene, a negative regulator of meristem identity [48], was notably suppressed in the shoot but induced in the root (Fig. 5, Table 3, Additional file 1: Table S1). This intriguing expression profile was verified by the qRT-PCR results (r = ~ 0.99) (Additional file 3: Table S3). Furthermore, we observed that the MADS-domain-carrying *VvSVP* gene was highly suppressed in all three tissues (Fig. 5). As predicted from the suppression status of the key flowering induction genes above, we found that some of the floral meristem identity genes were not induced. Although the floral meristem identity gene *FLOWERING LOCUS T* (*FT*) had no detectable transcripts in both NT and transgenic lines, ﻿*SUPPRESSOR OF OVEREXPRESSION OF CONSTANS 1* (*SOC1)* was expressed but not significantly differentially expressed. LFY was found expressed only in the shoot where it was likewise not differentially expressed. In grapevine, these meristem identity genes were expressed in the lateral branches after dormancy [49].

**Table 3:** Important DEGs predicted to carry both DELLA (*VvDELLA1*) and TALE (i.e. *KNAT1*) motifs and their expression correlation to *Vvgai1* in the shoot, leaf and root

Gene ID	Arabidopsis ID	Gene Name	Correlation to *Vvgai1*			Annotation
			Shoot	Leaf	Root	
GSVIVG01036145001	AT2G27550	*VvTFL1*	−0.725***		0.765***	*CENTRORADIALIS*
GSVIVG01033064001	AT5G50570	*VvSBP12*	−0.713***	−0.844***		Squamosa promoter-binding protein-like (SBP domain) transcription factor family protein
GSVIVG01033519001	AT2G42200	*SPL9*	−0.749***	−0.827***		squamosa promoter binding protein-like 9
GSVIVG01010522001	AT5G50570	*SPL13*	−0.73***			Squamosa promoter-binding protein-like (SBP domain) transcription factor family protein
GSVIVG01037598001	AT3G22190	*IQD5*		−0.964***		IQ-domain 5
GSVIVG01027429001	AT4G00820	*IQD17*		−0.826***		IQ-domain 17
GSVIVG01004809001	AT4G22140	*EBS*		−0.821***		PHD finger family protein / bromo-adjacent homology (BAH) domain-containing protein
GSVIVG01001269001	AT2G45190	*YABBY1*		−0.923***		Plant-specific transcription factor YABBY family protein
GSVIVG01027648001	AT2G45190	*YABBY1*		−0.851***		Plant-specific transcription factor YABBY family protein
GSVIVG01019399001	AT5G41410	*BEL1*			0.811***	POX (plant homeobox) family protein
GSVIVG01019043001	AT2G23760	*BLH4*		−0.781***	0.715***	BEL1-like homeodomain 4
GSVIVG01009589001	AT5G42630	*KAN4*	−0.73***	−0.916***	0.868***	Homeodomain-like superfamily protein
GSVIVG01035391001	AT3G13810	*AtIDD11*		−0.8***	0.889***	Indeterminate(ID)-domain 11
GSVIVG01010283001	AT2G02070	*AtIDD5*			0.93***	Indeterminate(ID)-domain 5
GSVIVG01010284001	AT2G02070	*AtIDD5*		−0.733***	0.983***	Indeterminate(ID)-domain 5
GSVIVG01011412001	AT5G15230	*GASA4*		−0.845***		GAST1 protein homolog 4
GSVIVG01035048001	AT5G49300	*GATA16*		−0.879***		GATA transcription factor 16
GSVIVG01017011001	AT4G32890	*GATA9*		−0.911***		GATA transcription factor 9
GSVIVG01019913001	AT4G37740	*AtGRF2*		−0.841***		growth-regulating factor 2
GSVIVG01027535001	AT2G36400	*GRF3*	−0.863***			growth-regulating factor 3
GSVIVG01038629001	AT5G53660	*AtGRF7*		−0.852***		growth-regulating factor 7

*pval ≤0.05; **pval ≤0.01; ***pval ≤0.001

### Transcription factor motif enrichment

Transcription factor enrichment analysis among the *Vvgai1*-correlated DEGs (r ≥ abs (0.70)) revealed a total of 17 significantly over-represented regulatory motifs (Additional file 4: Table S4). Of these the DELLA motif was the most over-represented in all three tissues: 45% of the suppressed DEGs in the shoot (49 out of 110), 37% of the suppressed DEGs in the leaf (269 out of 725) and 34% of the induced DEGs in the root (126 out of the 371). These amounted to 395 DELLA-bearing DEGs which were about 13% of the 2986 *Vvgai1*-correlated DEGs.

To assess the extent of possible co-regulation of DELLA with other transcription factors, we examined these 395 DELLA-bearing DEGs for the abundance of other regulatory motifs. Computational motif prediction based on PlantTFDB 4.0 [50] revealed that aside from the DELLA motif, at least two of the 16 motifs discussed earlier were present in any of these DEGs (Table 3, Additional file 5: Table S5), highlighting the complex nature of interdependency among transcriptional networks involving DELLAs. The most frequent motifs co-located with DELLA were gene families of TALE, TCP, INDETERMINDATE DOMAIN (IDD), AP2/ERF, and MYELOBLASTOSIS (MYB). TALE was the most frequent motif co-located with DELLA, sharing 71% (35 out of 49), 62% (167 out of 269) and 46% (59 out of 126) of the DELLA-enriched DEGs in the shoot, leaf and root, respectively (Additional file 5: Table S5). The remaining motifs were of lower frequency and tend to be tissue-specific. The class I and II TCP motifs, for example, were found in about 4–10% of the DELLA-enriched DEGs, most of which were in the leaf. The R2R3 MYB motif, as represented by three genes (*MYB15, MYB61, MYB88*) involved in stomatal movement and lignification [51–53], was co-located with DELLA in about 4–60% of the DELLA-enriched DEGs in the shoot, 4–15% in the leaf, and 2–15% in the root. The C2H2 zinc finger motif were represented by two IDD genes (*IDD5* and *IDD11*) and co-shared with the DELLA motif in 4–8% of the DEGs in the shoot, 5–8% in the leaf, and 2–17% in the root.

The DEGs with predicted DELLA-TALE motifs include notable meristem regulator genes in varying correlation to *Vvgai1* in the respective tissues. In the shoot, they included the suppressed FT-LIKE homologue *VvTFL1* (r = − 0.73) and *VvSBP12* (r = − 0.73)*, SPL13* (r = − 0.71), and *SPL9* (r = − 0.75) (Table 3). In the leaf, they included suppressed genes such as the protein-protein mediating calmudulin, *IQ-domain 5* (r = − 0.96), auxin-response factor 11 (r = − 0.93), flowering regulator *EARLY BOLTING IN SHORT DAYS* (r = − 0.82), leaf morphogenesis regulator *YABBY1* (r = − 0.92) and the homeodomain *BLH4* (r = − 0.78). In the root, they included induced genes *VvTFL1* (r = 0.76), *BEL1* (r = 0.81), an integument regulator *KANADI 4* (r = 0.84), and *IDD5* (r = 0.93) in the root (Table 3).

## Discussion

Global responses to DELLA-mediated regulation were previously reported but most were on annual model species, for a specific tissue or a specific developmental stage [21, 54, 55]. In this study, we analyzed RNA-Seq profiles in the shoot, leaf, and root of five transgenic GA-insensitive lines whose severity of mutant phenotypes corresponded well with the levels of transgene *Vvgai1* expression. By examining differential expression and correlation to *Vvgai1* in three tissues, we were able to dissect both global and tissue-specific regulation of DELLA, one of its characteristic regulatory features [56]. Our study has not only confirmed many of the previously reported GA- and DELLA-related genes and GO processes, but also found new DELLA targets and action modes, including the involvement of meristem regulators in co-regulating vine development and identification of several key flowering induction genes which responded to *Vvgai1* differently from annual species.

### DELLA’s impact on the expression of GA signaling genes

In agreement with the previous report [19], we found *VvDELLA2* abundantly expressed in all three tissues of the NT, followed by *VvDELLA1*, while the expression of *VvDELLA3* was generally low. Interestingly, the endogenous *VvDELLA*s were significantly induced, but varied from tissue to tissue, suggesting their regulatory interdependence and tissue-specific actions [19, 57]. Equally interesting was the observation that the *Vvgai1* (mutant DELLA) and induced endogenous DELLA transcripts in transgenic lines were not degraded as expected in a wildtype background. This was also in line with the observation of very low transcript levels of *VvSLY1a* and *VvSLY1b* (Fig. 3b), the two F-box genes that degrade DELLA through ubiquination [58]. Low *SLY1* expression was previously found associated with unusually high accumulation of *VvDELLA* and *VvGID1* transcripts [59]. We also noted that the putative DELLA-driven feedback induction of *VvGID1* [15] was tissue-specific as only one of the two *VvGID1* homologues (*VvGID1b)* exhibited a positively correlated change to *Vvgai1* and prominently in the leaf tissue. On the other hand, different homologues of GA biosynthesis components (GA20 oxidases and GA3 oxidases) were induced in the same manner as in annual [12, 21] and perennial species [10].

### DELLA and co-regulation networks in grapevine

The 2986 DEGs identified in this study represent a large number of enriched GO terms which overlapped in the three tissues in various degrees and response states, reflecting not only the breadth of GA signaling cascades [55] but also the dynamic coordination of biological functions within and between tissues [1, 60]. For example, one of the larger GO biological terms was related to meristem regulation and pattern-specification processes and largely enriched among the suppressed DEGs in the shoot and leaf. Included in the GO term were key meristem regulators of gene families such as GRFs, SPLs, SVPs, and genes such as *YABBY*, *AS1, STM* and *KNAT1*. In the root, however, the same GO term was enriched among the induced DEGs, including the auxin receptor *TIR1* and the major root growth and patterning regulators, the GRAS *SHORT ROOT* (*SHR), SCARECROW (SCR),* and the transcription factor *IDD5*.

Misregulations of the meristem-related developmental processes in the transgenic vines was apparent all the more by the fact that numerous classes of homeodomain transcription factors were generally in a suppressed state in the sampled tissues, including the class I KNOX transcription factors which interact with GA pathway genes in the maintenance of SAM [21, 37, 39]. In *Arabidopsis*, the class I KNOX genes *STM* and *KNAT1* critically coordinate proper SAM growth by properly localizing the hormones cytokinin and GA [39, 61, 62]. Transcriptome scans of GA-insensitive *Arabidopsis* mutants seemed to suggest that both *STM* and *KNAT1* were not particularly expressed at the early stage [15, 55, 63] as much as during the adult flowering stage [54, 64]. Our discovery of a large number of key homeodomain transcription factors among the responsive DEGs at the early growth stages of the GA-insensitive grapevines highlights the unique organ development process in a grapevine shoot – a separate vegetative and reproductive meristem (i.e. anlagen) in the same apical meristem – and the complex regulation of vegetative and reproductive phase transition it entails.

Such complex regulation was also reflected by 17 large and diverse transcription factor motifs statistically over-represented among the DEGs. Of which, DELLA was the largest, found in about 30% of the total DEGs, followed by motifs of several meristem regulators such as TALE, TCP, MYB and IDD, and stress-response regulators, WRKY, NAC and bHLH. Many of these motifs were co-located with DELLA motifs in DEGs, providing opportunities for DELLA to interact with other transcription factors to co-regulate diverse biological processes [7, 65]. Indeed, in the DELLA-motif-bearing DEGs, motifs of several important regulators were present in varying enrichment in three tissues. In the root, the motifs of AP2/ERF, MYB and IDD were relatively more enriched. The enrichment of IDD motif was notable because the IDD5 homologues were recently shown to act as a transcriptional scaffold in the competitive interaction between *SCR* and *DELLA* in mediating root growth [66]. On the other hand, in the suppressed DELLA-bearing DEGs of the shoot and leaf, the motifs of TALE, TCP and MYB were more enriched. TALE was particularly notable because it was the most frequent motif being co-present with DELLA. The putative DELLA-TALE bearing gene families included genes affecting germination (Nuclear Factors Y, IDDs), growth (GATAs), internode elongation (GRFs), shoot and leaf expansion (YABBY, BEL), as well as floral induction phase-change (SPLs*, VvTFL1*) pathways. The discoveries of these diverse transcription regulators and co-presences of their motifs with DELLA in the DEGs provide a potential explanation for how GA-DELLA signaling could impact so many different aspects of plant growth and development.

### DELLA and root growth in grapevine

One interesting phenotypic change of the transgenic vines was an enlarged root which had poor rooting abilities (data not shown). The root tissue had the largest number of DEGs, compared with the shoot and leaf tissue in the transgenic lines. DELLA motif was statistically over-represented among the induced DEGs. Among these induced DELLA-motif-bearing DEGs were *SHR*, *SCR* and *SCL3*, which are GRAS genes involved in root maintenance and patterning [67]. In the transcriptome profiling of a transgenic GA insensitive poplar, the GRAS *SCR* was also among the genes found induced [68]. However, we observed numerous cyclin genes, D-type cyclins in particular, in a suppressed state in the leaf and root of our transgenic vines, while they were induced in transgenic poplar [68]. The D-type cyclins are mutual activators of SHR-SCR complex [69] and cytokinin [70] regulatory networks for formative cell divisions in the apical meristem, and formation of lateral organs [71]. Suppression of these cyclins in the present study may compromise their interaction with the SHR-SCR complex and cytokinin, which may explain the observed poor rooting ability in our study, in a contrast to the vigorous roots observed in transgenic poplar.

We deduce that cytokinin was diminished in our transgenic lines based on the relatively low transcript levels of its biosynthesis (i.e. *IPT2* and *IPT3*), signal reception (e.g. *AHK4*), and response regulators (type B RR) components. The enlarged roots were likely the manifestation of a very low cytokinin level in the transition zone region, where cytokinin is usually kept relatively higher than auxin to favor cell differentiation and establishment of the size homeostasis of root meristems [72]. In contrast, a root tip usually maintains a high level of auxin that favors cell division needed for maintenance of the stem cell niche, which seemed to be the case in our transgenic vines as indicated by the induced state of auxin biosynthesis (i.e. *YUCCA*), signal receptor (i.e. *TIR1*), transporter (i.e. PINs) and response factors (i.e. ARFs). Critically, we observed that *SHY2*, the key negative regulator governing auxin:cytokinin crosstalk and balance [73], was suppressed and negatively correlated to *Vvgai1* in the shoot and root. We were unable to find and verify if *SHY2* was likewise suppressed in the microarray-based study of the transgenic GA insensitive poplar [68]. However, our transgenic lines seemed chronically deficient in cytokinin and high in auxin from the expression profiles discussed above. The enlarged roots in our transgenic vines were likely due to low levels of cytokinin and thus reduced levels of cell differentiation and proper control of root meristem size. In parallel, the disrupted balance between auxin:cytokinin might result in more accumulation of auxin which can lead to poor rooting ability as previously observed in *Arabidopsis* [74].

### DELLA and grapevine flowering-induction

The induced responses of *VvCO* and *VvCOL1* to *Vvgail* discovered in this study was in a sharp contrast to the findings that *AtCO* was suppressed by DELLA in *Arabidopsis* [47]. Recent study showed that *VvCO* and *VvCOL1* positively regulated lateral bud induction and dormancy of grapevine; but in contrast to the *CO* and *COL1* in *Arabidopsis*, the diurnal oscillation of *VvCO* and *VvCOL1* was less affected by continuous light and had a higher amplitude that instead peaked at dawn [75]. Since it was shown that lower temperature favors tendril over inflorescence in grapevine [76] and GA signaling exhibited diurnal periodicity in *Arabidopsis* [77], GA and the photoperiod pathway genes in grapevine might have been adaptively modified to perceive warmer temperatures and light intensity instead, and less of daylength as dominant stimuli for flower induction [76].

Similar to GA-insensitive *Arabidopsis* [45], expression of several *VvSPL* genes were suppressed in the shoot and leaf of our transgenic lines. The GA-insensitive *Arabidopsis* was late-flowering because DELLA sequestered the *SPL* regulators and essentially prolonged the juvenile phase. However, the distinction between juvenile and adult phases is less defined in grapevine and obscured by the simultaneous presence of vegetative and reproductive meristems in the same shoot [78]. Recent transcriptome survey of tendril and inflorescence development revealed that *VvSPL*s were particularly expressed in the early stages of both tendril and inflorescence development [79], suggesting that the equivalent of phase-transition in grapevine takes place when the anlagen differentiates towards tendril or inflorescence formation. At least one of the *VvSPL*s, *VvSBP12,* was reported to carry the regulation motif of miRNA156/172 [44], which raises the possibility of the role of miRNAs in grapevine shoot architecture and anlagen developmental processes. *VvSBP12’s* homologues in annual model plants mediate numerous phase-related developmental processes, including initiation of the first true leaves in *Arabidopsis* (*AtSPL13* [80]), grain enlargement (*OsSPL16* [81]), and ear glume development in maize (*TGA1* [82]). The suppression of *VvSPL* genes in this study might explain the observed progressive severity of tendril-less nodes correlated with high levels of *Vvgai1* expression. We noted that HEN1, a dicer-like gene involved in miRNA metabolism [46] was induced in the shoot and leaf. HEN1, however, was suppressed in the root, while another dicer homologue, CARPEL FACTORY (CAF) was not correlated to *Vvgai1*, suggesting the existence of complex interactions among DELLA, SPLs and miRNA.

We found that the gene *VvSVP,* a MADS box gene also involved in phase-transition dependent flowering in *Arabidopsis* [45, 83], was significantly suppressed in leaf and shoot tissues. The suppressed expression of *VvSVP* and *VvSBP12* in the transgenic plants was in line with the observed delayed tendril emergence and a progressive disruption in shoot phyllotaxy. This is also supported by the fact that *VvSVP* was considered as a positive regulator of floral transition in grapevine [13, 79, 84], in contrast to being a negative regulator in *Arabidopsis* [83].

Interestingly, we also discovered an antagonistic flowering gene, *VvTFL1*, induced in the root, suppressed in the shoot and not expressed in the leaf. Since *VvTFL1* is cell mobile [85], the induced *VvTFL1* in the root could migrate and function as an antagonist of the meristem identity gene *LFY* and floral integrator *VvFT* in grapevine shoot. This finding may suggest that roots are involved in the over-all regulation of flowering in grapevine via GA-DELLA signaling cascade.

## Conclusion

Grapevine as a perennial species shared a similar DELLA-centered feedback mechanism as annual species for maintaining GA homeostasis and controlling plant development and growth through intricate interactions of DELLAs with numerous and specialized transcription factors. However, some of these interaction outcomes could be very different between perennial and annual species, as illustrated in this study that the expression behaviors of certain flowering induction and development pathway genes in the grapevine were in sharp contrast with that of annual species. It was interesting to observe that homeodomain transcription factors seemed to play important roles in coordinating vegetative and reproductive transition in grapevine in which both vegetative and reproductive meristems simultaneously co-exist in the same shoot.

## Methods

### Expression cassette and transformation

A binary vector construct, named as pVv::VvGAI^L38H^, was used in this study, containing a modified 5.1 kb-long fragment of the grape *VvGAI* genomic sequence cloned from *V. vinifera* ‘Pinot Noir’. The modified *VvGAI* genomic sequence has a 2171-bp sequence upstream of the ATG start codon, a 1773-bp coding sequence with an introduced point mutation, which results in replacing a leucine amino acid (L) by a histidine (H) at the amino acid position 38 in the DELLA domain of the encoded GAI protein, and a 1157-bp sequence downstream of the stop codon. Other detail information about the construct was previously reported [14].

The Ralph M. Parsons Plant Transformation Facility at the University of California, Davis (https://ptf.ucdavis.edu/services) generated the transgenic grapevines through *Agrobacterium*-based transformation in the *V. vinifera* ‘Thompson Seedless’ background for this study. Ten transgenic *Vvgai1* vines were generated and five of the representative ones were used in this study after preliminary evaluation (Table 1). A non-transgenic *V. vinifera* ‘Thompson Seedless’ was provided as a control.

### Propagation of transgenic grapevines and phenotyping

Freshly received transgenic grapevines were potted in 4-in. pots using Promix Biofungicide media (Premiere Horticulture, Canada). Green stem cuttings from individual transgenic lines were used to generate multiple clones for subsequent experiments. Vines were maintained in 1-gal pots in a greenhouse by applying routine vine management practices.

Phenotypic traits, such as plant height, internode length and tendril distribution pattern were observed from five biological replicates of the transgenic lines and NT. In the case of leaf weight, the measurements were from 5 to 10 pooled leaves in each biological replicate. Similarly, total chlorophyll content was assessed from pooled leaf discs of five expanded leaves of each vine following an acetone extraction method [86]. Root characteristics were assessed from depotted pots after 5-month of growth in the greenhouse.

### RNA-Seq library preparation and sequence reads processing

All tissues for RNA-Seq profiling were collected from 5-month-old vine clones of individual transgenic lines. Duplicated samples from at least two clonal vines were collected for each transgenic line and the non-transgenic control. Three types of tissues were taken. The shoot tissue sample contained 1-3 cm shoot tips of main branches, comprised of the apical region and two young, unfolding leaves. The leaf tissue sample was pooled 5th and 6th expanded leaves from the top and the root was the pool of several 3 cm-clippings of roots from depotted plants. All samples were taken fresh, flash frozen and stored at − 80 °C until further processing.

RNA extraction and RNA-Seq library preparation were performed as previously described [87]. RNA-Seq libraries were multiplexed for paired-end 2 × 100-bp (root and shoot tissue samples) or single-end 100-bp (leaf tissue samples) sequencing using Illumina HiSeq 2000 at the Cornell University Biotechnology Resource Center, Ithaca, NY.

Sequence reads of each RNA-Seq library were pre-processed using Trimmomatic (Illumina, San Diego, CA, USA) and FastQC. The artifact-free sequences were then individually aligned to the *Vitis* reference genome (12X *V. vinifera*, Phytozome ver. 12) using Tophat2, following the workflow for splice-aware RNAseq alignment (http://ccb.jhu.edu/software/tophat/manual.shtml).

### Digital RT-PCR validation

Thirty genes were selected for digital RT-PCR verification of the RNA-Seq gene expression. cDNAs were synthesized from the same mRNAs processed for RNA-Seq libraries. The Qiagen multiplex PCR plus kit was used for the 1st round of amplification of 150–200 bp fragments in individual genes. For each tissue sample, four multiplex reactions were performed to cover 40 different regions in 30 genes. For the 2nd round PCR amplification, the Illumina Nextera dual-indexing primer system was used to barcode each individual sample and the PCR reaction was purified by AmpureXP and eluted in 10 μl water. Equal amount of DNA from individual libraries was pooled together for Illumina HighSeq sequencing (2 × 150 paired end).

Three sequence fragments each with an allele-specific SNP were amplified to confidently distinguish the transgenic *GAI* allele (*Vvgai1*, [14]) from non-transgenic one (*VvGAI1*, GSVIVG01011710001 [19]) in the ‘Thompson Seedless’ background. The ratios of *Vvgai1*/*VvGAI1* in the three regions were then averaged and the mean ratio was used to reflect the relative qRT-PCR expression levels of the transgene *Vvgai1* in a tissue. The mean ratios derived from the qRT-PCR data were further used for a correlation analysis of the RNA-Seq expression levels of individual endogenous genes with that of *Vvgai1* across different transgenic vines.

### Differential expression analysis

For each tissue, we constructed and analyzed 12 RNA-Seq library samples: five transgenic lines and one non-transgenic Thompson Seedless, each with two biological replicates. The shoot library samples were single-end sequenced while the other two tissues were paired-end sequenced.

The abundance of each read was determined by HTseq [88] and differential expression analysis was done using EdgeR [89], following the analysis workflow described in our previous work [28]. To focus on the genes which were most likely affected by *Vvgai1*, we included in the further analysis only those differentially expressed genes (1.5x-fold changes at FDR ≤ 0.01) identified in the strongest transgenic line G02 and also in at least one other transgenic line when compared with the non-transgenic control (NT). We used count per million mapped reads (CPM) as the normalized expression unit in assessing the expression level of a gene.

### Functional analysis

Gene Ontology (GO) analyses were done for the DEGs correlated to *Vvgai1* (r > = 0.50) in individual tissues using Plant MetGenMAP [90]. To simplify pattern interpretation, the resulting lists of enriched GO terms (FDR ≤ 0.10) were clustered into similar functions using REVIGO [91]. To assess the relative responses of endogenous genes to the transgenic *Vvgai1* expression, pairwise correlation was computed between the expression variation of each DEG among the transgenic lines with the relative expression level of *Vvgai1* (ratio of *Vvgail1*/*VvGAI1*) in each tissue. The average correlation indices of the genes in a particular enriched biological GO term was then taken as the relative gauge of association to *Vvgai1*. Transcription factor enrichment and putative pairwise regulator-target interactions were facilitated by using PlantTFDB 4.0 and PlantRegMap [50]. Gene annotation, cross-referencing, and sequence homology analyses were conducted using TAIR [92], Phytozome 12 [93], PlantTFDB 4.0 and PlantMetGenMap [90]. Whenever possible, we used the gene names reported in relevant grapevine research. Otherwise we followed the nomenclature indicated in the Genoscope’s 12X *V. vinifera* genome database (http://www.genoscope.cns.fr/externe/GenomeBrowser/Vitis/) in conjunction with the TAIR database (https://www.arabidopsis.org/). For providing further clarity and avoiding potential confusion, whenever appropriate we added a prefix “*Vv*” to a gene name referred in the main text as presented in Additional file 1: Table S1.

## Additional files

Additional file 1: **Table S1.** A list of DEGs and their expression correlation to Vvgai1 in the shoot, leaf and root tissue. (XLSX 530 kb)

Additional file 2: **Table S2.** A summary of the uniquely induced and suppressed genes (XLSX 11 kb)

Additional file 3: **Table S3.** Pearson correlation (r) of standardized RNA-Seq and digital qRT-PCR expressions for a selection of 30 genes in the transgenic lines. (XLSX 11 kb)

Additional file 4: **Table S4.** Transcription factor motifs significantly over-represented among the DEGs correlated to *Vvgai1* (r ≥ 0.70) in the shoot, leaf and root tissues. (XLSX 13 kb)

Additional file 5: **Table S5.** A list of the 395 DELLA-bearing DEGs with co-presences of other TF motifs predicted. (XLSX 67 kb)

## Abbreviations

ARF: Auxin Response Factor
BEL: Bellringer
CAF: Carpel factory
CKX: Cytokinin oxidase
CO: Constans
CPM: Count per million reads aligned
FT: Flowering locus 1
GA: Gibberellic acid
Ga20ox: GA20 oxidase
Ga3ox: GA3 oxidase
GAI: Gibberellic acid-insensitive
GATA: GATA-binding
GID1: GA insensitive dwarf1
GO: Gene Ontology
GRF: Growth regulating factor
HD-ZIP: Homeodomain-leucine zipper
IDD: Indeterminate domain
IPT: Isopentenyltransferase
KNAT1: Knotted-like from *Arabidopsis thaliana*1
KNOX: Knotted1-like
MYB: Myeloblastosis
NAC: Nam/ataf1,2/cuc2
NT: Non-transgenic
PIN: Pin-formed
SAM: Shoot apical meristem
SCR: Scarecrow
SHR: Short root
SHY2: Short hypocotyl 2
SOC1: Suppressor of overexpression of constans 1
SPL: Squamosa-promoter binding protein-like
STM: Shoot meristemless
SVP: Short vegetative phase
TALE: Three-amino-acid-loop-extension
TCP: Tb1-Cyc-Pcf
TFL1: Terminal flower 1 or Centroradialis
TGA1: Teosinte glume architecture
TIR1: Transport inhibitor response1
Vvgai1: Grapevine gibberellic acid-insensitive1
WOX: Wuschelrelated homeobox

## References

[1] Dayan J. Gibberellin Transport. In: Annual Plant Reviews: The Gibberellins; 2016. p. 95–120. 10.1002/9781119210436.ch4.

[2] Murase K, Hirano Y, Sun TP, Hakoshima T. Gibberellin-induced DELLA recognition by the gibberellin receptor GID1. Nature. 2008;456:459–63. 10.1038/nature07519. 19037309

[3] Hedden P. The genes of the green revolution. Trends Genet. 2003;19:5–9. 10.1016/S0168-9525(02)00009-4. 12493241

[4] Peng J, Carol P, Richards DE, King KE, Cowling RJ, Murphy GP, et al. The Arabidopsis GAI gene defines a signaling pathway that negatively regulates gibberellin responses. Genes Dev. 1997;11:3194–205. 10.1101/gad.11.23.3194. 10.1101/gad.11.23.3194 PMC316750 9389651

[5] Fleet CM, Sun TP. A DELLAcate balance: the role of gibberellin in plant morphogenesis. Curr Opin Plant Biol. 2005;8:77–85. 10.1016/j.pbi.2004.11.015. 15653404

[6] Sun T. Gibberellin-GID1-DELLA: a pivotal regulatory module for plant growth and development. Plant Physiol. 2010;154:567–70. 10.1104/pp.110.161554. PMC2949019 20921186

[7] Van De Velde K, Ruelens P, Geuten K, Rohde A, Van Der Straeten D. Exploiting DELLA signaling in cereals. Trends Plant Sci. 2017;22:880–93. 10.1016/j.tplants.2017.07.010. 28843766

[8] Busov VB. Activation tagging of a dominant gibberellin catabolism gene (GA 2-oxidase) from poplar that regulates tree stature. Plant Physiol. 2003;132:1283–91. 10.1104/pp.103.020354. PMC167068 12857810

[9] Schomburg FM, Bizzell CM, Lee DJ, Zeevaart AD, Amasino RM. Overexpression of a novel class of gibberellin 2-oxidases decreases gibberellin levels and creates dwarf plants. Plant Cell. 2003;15:151–63. 10.1105/tpc.005975. 10.1105/tpc.005975 PMC143488 12509528

[10] Busov V, Meilan R, Pearce DW, Rood SB, Ma C, Tschaplinski TJ, et al. Transgenic modification of gai or rgl1 causes dwarfing and alters gibberellins, root growth, and metabolite profiles in Populus. Planta. 2006;224:288–99. 10.1007/s00425-005-0213-9. 16404575

[11] Dill A, Jung HS, Sun TP. The DELLA motif is essential for gibberellin-induced degradation of RGA. Proc Natl Acad Sci. 2001;98:14162–7. 10.1073/pnas.251534098. 10.1073/pnas.251534098 PMC61185 11717468

[12] Boss PK, Thomas MR. Association of dwarfism and floral induction with a grape “green revolution” mutation. Nature. 2002;416:847–50. 10.1038/416847a. 10.1038/416847a 11976683

[13] Li-Mallet A, Rabot A, Geny L. Factors controlling inflorescence primordia formation of grapevine: their role in latent bud fruitfulness? A review. Botany. 2016;94:147–63. 10.1139/cjb-2015-0108.

[14] Zhong GY, Yang Y. Characterization of grape gibberellin Insensitive1 mutant alleles in transgenic Arabidopsis. Transgenic Res. 2012;21:725–41. 10.1007/s11248-011-9565-z. 10.1007/s11248-011-9565-z 22038449

[15] Zentella R, Zhang Z-L, Park M, Thomas SG, Endo A, Murase K, et al. Global analysis of DELLA direct targets in early gibberellin signaling in *Arabidopsis*. Plant Cell. 2007;19:3037–57. 10.1105/tpc.107.054999. PMC2174696 17933900

[16] Davière JM, Achard P. A Pivotal Role of DELLAs in Regulating Multiple Hormone Signals. Molecular Plant. 2016;9:10–20. 10.1016/j.molp.2015.09.011. 10.1016/j.molp.2015.09.011 26415696

[17] Srinivasan C, Mullins MG. Flowering in Vitis: conversion of tendrils into inflorescences and bunches of grapes. Planta. 1979;145:187–92. 10.1007/BF00388716. 24317675

[18] Gerrath J, Posluszny U, Melville L. Taming the wild grape: botany and horticulture in the vitaceae. Cham: Springer International Publishing; 2015. 10.1007/978-3-319-24352-8.

[19] Acheampong AK, Hu J, Rotman A, Zheng C, Halaly T, Takebayashi Y, et al. Functional characterization and developmental expression profiling of gibberellin signalling components in Vitis vinifera. J Exp Bot. 2015;66:1463–76. 10.1093/jxb/eru504. 10.1093/jxb/eru504 PMC4339604 25588745

[20] Yamaguchi S, Kamiya Y. Gibberellin biosynthesis: its regulation by endogenous and environmental signals. Plant Cell Physiol. 2000;41:251–7. 10.1093/pcp/41.3.251. 10805587

[21] Sun TP. Gibberellin metabolism, perception and signaling pathways in Arabidopsis. Arabidopsis Book. 2008;6:e0103. 10.1199/tab.0103. 10.1199/tab.0103 PMC3243332 22303234

[22] Omidbakhshfard MA, Proost S, Fujikura U, Mueller-Roeber B. Growth-Regulating Factors (GRFs): A Small Transcription Factor Family with Important Functions in Plant Biology. 2015. 10.1016/j.molp.2015.01.013. 25620770

[23] Horiguchi G, Kim GT, Tsukaya H. The transcription factor AtGRF5 and the transcription coactivator AN3 regulate cell proliferation in leaf primordia of Arabidopsis thaliana. Plant J. 2005;43:68–78. 10.1111/j.1365-313X.2005.02429.x. 10.1111/j.1365-313X.2005.02429.x 15960617

[24] Yoshimitsu Y, Tanaka K, Fukuda W, Asami T, Yoshida S, Hayashi K, et al. Transcription of DWARF4 plays a crucial role in auxin-regulated root elongation in addition to Brassinosteroid homeostasis in Arabidopsis thaliana. PLoS One. 2011;6:e23851. 10.1371/journal.pone.0023851. PMC3166115 21909364

[25] Gao X, Zhang Y, He Z, Fu X. Gibberellins. In: Hormone Metabolism and Signaling in Plants; 2017. p. 107–60. 10.1016/B978-0-12-811562-6.00004-9.

[26] Nakamoto D. Inhibition of Brassinosteroid biosynthesis by either a dwarf4 mutation or a Brassinosteroid biosynthesis Inhibitor rescues defects in tropic responses of hypocotyls in the Arabidopsis mutant nonphototropic hypocotyl 4. Plant Physiol. 2006;141:456–64. 10.1104/pp.105.076273. PMC1475450 16632588

[27] Nicolas M, Cubas P. TCP factors: new kids on the signaling block. Curr Opin Plant Biol. 2016;33:33–41. 10.1016/j.pbi.2016.05.006. 27310029

[28] Arro J, Cuenca J, Yang Y, Liang Z, Cousins P, Zhong GY. A transcriptome analysis of two grapevine populations segregating for tendril phyllotaxy. Hortic Res. 2017;4:17032. 10.1038/hortres.2017.32. 10.1038/hortres.2017.32 PMC5506248 28713572

[29] Moore M, Vogel MO, Dietz KJ. The acclimation response to high light is initiated within seconds as indicated by upregulation of AP2/ERF transcription factor network in Arabidopsis thaliana. Plant Signal Behav. 2014;9:1–4. 10.4161/15592324.2014.976479. PMC4622746 25482793

[30] Bolt S, Zuther E, Zintl S, Hincha DK, Schmulling T. ERF105 is a transcription factor gene of Arabidopsis thaliana required for freezing tolerance and cold acclimation. Plant Cell Environ. 2017;40:108–20. 10.1111/pce.12838. 27723941

[31] Wang K-C, Liu X, Chen C-Y, Wu K. Roles of Phytochrome-interacting Factors in Light Signaling. J Plant Biochem Physiol. 2013;1. 10.4172/2329-9029.1000e114.

[32] Hedden P, Sponsel V. A century of gibberellin research. J Plant Growth Regul. 2015;34:740–60. 10.1007/s00344-015-9546-1.. 10.1007/s00344-015-9546-1 PMC4622167 26523085

[33] Tian Q. Arabidopsis SHY2/IAA3 Inhibits Auxin-Regulated Gene Expression. Plant Cell. 2002;14:301–19. 10.1007/s00344-015-9546-1.10.1105/tpc.010283. 10.1105/tpc.010283 PMC152914 11884676

[34] Gray WM, Kepinski S, Rouse D, Leyser O, Estelle M. Auxin regulates SCFTIR1-dependent degradation of AUX/IAA proteins. Nature. 2001;414:271–6. 10.1038/35104500. 11713520

[35] Wang Y, Shen W, Chan Z, Wu Y. Endogenous cytokinin overproduction modulates ROS homeostasis and decreases salt stress resistance in Arabidopsis thaliana. Front Plant Sci. 2015;6:1-69. 10.3389/fpls.2015.01004. PMC4652137 26635831

[36] Feng J, Shi Y, Yang S, Zuo J. Cytokinins. In: Hormone Metabolism and Signaling in Plants; 2017. p. 77–106. 10.1016/B978-0-12-811562-6.00003-7.

[37] Hay A, Craft J, Tsiantis M. Plant hormones and homeoboxes: bridging the gap? BioEssays. 2004;26:395–404. 10.1002/bies.20016. 10.1002/bies.20016 15057937

[38] Byrne ME. Phyllotactic pattern and stem cell fate are determined by the Arabidopsis homeobox gene BELLRINGER. Development. 2003;130:3941–50. 10.1242/dev.00620. 12874117

[39] Jasinski S, Piazza P, Craft J, Hay A, Woolley L, Rieu I, et al. KNOX action in Arabidopsis is mediated by coordinate regulation of cytokinin and gibberellin activities. Curr Biol. 2005;15:1560–5. 10.1016/j.cub.2005.07.023. 10.1016/j.cub.2005.07.023 16139211

[40] Lincoln C, Long J, Yamaguchi J, Serikawa K, Hake S. A knotted1-like homeobox gene in Arabidopsis is expressed in the vegetative meristem and dramatically alters leaf morphology when overexpressed in transgenic plants. Plant Cell. 1994;6:1859–76. 10.1105/tpc.6.12.1859. PMC160567 7866029

[41] Rutjens B, Bao D, van Eck-Stouten E, Brand M, Smeekens S, Proveniers M. Shoot apical meristem function in Arabidopsis requires the combined activities of three BEL1-like homeodomain proteins. Plant J. 2009;58:641–54. 10.1111/j.1365-313X.2009.03809.x. 19175771

[42] Stahl Y, Simon R. Plant primary meristems: shared functions and regulatory mechanisms. Curr Opin Plant Biol. 2010;13:53–8. 10.1016/j.pbi.2009.09.008. 10.1016/j.pbi.2009.09.008 19836993

[43] Ariel FD, Manavella PA, Dezar CA, Chan RL. The true story of the HD-Zip family. Trends Plant Sci. 2007;12:419–26. 10.1016/j.tplants.2007.08.003. 10.1016/j.tplants.2007.08.003 17698401

[44] Belli Kullan J, Lopes Paim Pinto D, Bertolini E, Fasoli M, Zenoni S, Tornielli GB, et al. miRVine: A microRNA expression atlas of grapevine based on small RNA sequencing. BMC Genomics. 2015;16:393. 10.1186/s12864-015-1610-5. PMC4434875 25981679

[45] Yu S, Galvão VC, Zhang Y-CY-C, Horrer D, Zhang T-QT-Q, Hao Y-HY-H, et al. Gibberellin regulates the Arabidopsis floral transition through miR156-targeted SQUAMOSA PROMOTER BINDING-LIKE transcription factors. Plant Cell. 2012;24:3320–32. 10.1105/tpc.112.101014. PMC3462634 22942378

[46] Park W, Li J, Song R, Messing J, Chen X. CARPEL FACTORY, a Dicer Homolog, and HEN1, a Novel Protein, Act in microRNA Metabolism in Arabidopsis thaliana. Curr Biol. 2002;12:1484–95. 10.1016/s0960-9822(02)01017-5 PMC5137372 12225663

[47] Xu F, Li T, Xu PB, Li L, Du SS, Lian HL, et al. DELLA proteins physically interact with CONSTANS to regulate flowering under long days in Arabidopsis. FEBS Lett. 2016;590:541–9. 10.1002/1873-3468.12076. 26801684

[48] Boss PK, Sreekantan L, Thomas MR. A grapevine TFL1 homologue can delay flowering and alter floral development when overexpressed in heterologous species. Funct Plant Biol. 2006;33:31. 10.1071/FP05191. 10.1071/FP05191 32689212

[49] Carmona MJ, Chaib J, Martinez-Zapater JM, Thomas MR. A molecular genetic perspective of reproductive development in grapevine. J Exp Bot. 2008;59:2579–96. 10.1093/jxb/ern160. 10.1093/jxb/ern160 18596111

[50] Jin J, Tian F, Yang D-C, Meng Y-Q, Kong L, Luo J, et al. PlantTFDB 4.0: toward a central hub for transcription factors and regulatory interactions in plants. Nucleic Acids Res. 2017;45:D1040–5. 10.1093/nar/gkw982. 10.1093/nar/gkw982 PMC5210657 27924042

[51] Feller A, Machemer K, Braun EL, Grotewold E. Evolutionary and comparative analysis of MYB and bHLH plant transcription factors. Plant J. 2011;66:94–116. 10.1111/j.1365-313X.2010.04459.x. 10.1111/j.1365-313X.2010.04459.x 21443626

[52] Huang D, Wang S, Zhang B, Shang-Guan K, Shi Y, Zhang D, et al. A Gibberellin-Mediated DELLA-NAC Signaling Cascade Regulates Cellulose Synthesis in Rice. Plant Cell. 2015;27:1681–96. 10.1105/tpc.15.00015. 10.1105/tpc.15.00015 PMC4498200 26002868

[53] Chezem WR, Memon A, Li F-S, Weng J-K, Clay NK. SG2-Type R2R3-MYB Transcription Factor MYB15 Controls Defense-Induced Lignification and Basal Immunity in Arabidopsis. Plant Cell. 2017;29:1907–26. 10.1105/tpc.16.00954. 10.1105/tpc.16.00954 PMC5590497 28733420

[54] Hou X, Hu W-W, Shen L, Lee LYC, Tao Z, Han J-H, et al. Global Identification of DELLA Target Genes during Arabidopsis Flower Development. PLANT Physiol. 2008;147:1126–42. 10.1104/pp.108.121301. 10.1104/pp.108.121301 PMC2442519 18502975

[55] Claeys H, De Bodt S, Inzé D. Gibberellins and DELLAs: central nodes in growth regulatory networks. Trends Plant Sci. 2014;19:231–9. 10.1016/j.tplants.2013.10.001. 24182663

[56] Daviere J-M, Achard P. Gibberellin signaling in plants. Development. 2013;140:1147–51. 10.1242/dev.087650. 23444347

[57] Grimplet J, Agudelo-Romero P, Teixeira RT, Martinez-Zapater JM, Fortes AM. Structural and Functional Analysis of the GRAS Gene Family in Grapevine Indicates a Role of GRAS Proteins in the Control of Development and Stress Responses. Front Plant Sci. 2016;7. 10.3389/fpls.2016.00353. 10.3389/fpls.2016.00353 PMC4811876 27065316

[58] Dill A, Thomas SG, Hu J, Steber CM, Sun T-P. The Arabidopsis F-Box Protein SLEEPY1 Targets Gibberellin Signaling Repressors for Gibberellin-Induced Degradation. Plant Cell. 2004;16:1392–405. 10.1105/tpc.020958. 10.1105/tpc.020958 PMC490034 15155881

[59] Acheampong AK, Zheng C, Halaly T, Giacomelli L, Takebayashi Y, Jikumaru Y, et al. Abnormal Endogenous Repression of GA Signaling in a Seedless Table Grape Cultivar with High Berry Growth Response to GA Application. Front Plant Sci. 2017;8:850. 10.3389/fpls.2017.00850. 10.3389/fpls.2017.00850 PMC5442209 28596775

[60] Voß U, Bishopp A, Farcot E, Bennett MJ. Modelling hormonal response and development. Trends Plant Sci. 2014;19:311–9. 10.1016/j.tplants.2014.02.004. 10.1016/j.tplants.2014.02.004 PMC4013931 24630843

[61] Hay A, Kaur H, Phillips A, Hedden P, Hake S, Tsiantis M. The Gibberellin Pathway Mediates KNOTTED1-Type Homeobox Function in Plants with Different Body Plans. Curr Biol. 2002;12:1557–65. 10.1016/S0960-9822(02)01125-9. 10.1016/s0960-9822(02)01125-9 12372247

[62] Ikezaki M, Kojima M, Sakakibara H, Kojima S, Ueno Y, Machida C, et al. Genetic networks regulated by ASYMMETRIC LEAVES1 ( AS1 ) and AS2 in leaf development in Arabidopsis thaliana : KNOX genes control five morphological events. Plant J. 2010;61:70–82. 10.1111/j.1365-313X.2009.04033.x. 10.1111/j.1365-313X.2009.04033.x 19891706

[63] Locascio A, Blázquez MA, Alabadí D. Genomic Analysis of DELLA Protein Activity. Plant Cell Physiol. 2013;54:1229–37. 10.1093/pcp/pct082. 10.1093/pcp/pct082 23784221

[64] Cao D, Cheng H, Wu W, Soo HM, Peng J. Gibberellin Mobilizes Distinct DELLA-Dependent Transcriptomes to Regulate Seed Germination and Floral Development in Arabidopsis. Plant Physiol. 2006;142:509–25. 10.1104/pp.106.082289. 10.1104/pp.106.082289 PMC1586041 16920880

[65] Davière J-M, de Lucas M, Prat S. Transcriptional factor interaction: a central step in DELLA function. Curr Opin Genet Dev. 2008;18:295–303. 10.1016/j.gde.2008.05.004. 10.1016/j.gde.2008.05.004 18590820

[66] Yoshida H, Ueguchi-Tanaka M. DELLA and SCL3 balance gibberellin feedback regulation by utilizing INDETERMINATE DOMAIN proteins as transcriptional scaffolds. Plant Signal Behav. 2014;9:e29726. 10.4161/psb.29726. 10.4161/psb.29726 PMC4205140 25763707

[67] Heo J-O, Chang KS, Kim IA, Lee M-H, Lee SA, Song S-K, et al. Funneling of gibberellin signaling by the GRAS transcription regulator SCARECROW-LIKE 3 in the Arabidopsis root. Proc Natl Acad Sci. 2011;108:2166–71. 10.1073/pnas.1012215108. 10.1073/pnas.1012215108 PMC3033297 21245304

[68] Gou J, Strauss SH, Tsai CJ, Fang K, Chen Y, Jiang X, et al. Gibberellins Regulate Lateral Root Formation in Populus through Interactions with Auxin and Other Hormones. Plant Cell. 2010;22:623–39. 10.1105/tpc.109.073239. 10.1105/tpc.109.073239 PMC2861444 20354195

[69] Sozzani R, Cui H, Moreno-Risueno MA, Busch W, Van Norman JM, Vernoux T, et al. Spatiotemporal regulation of cell-cycle genes by SHORTROOT links patterning and growth. Nature. 2010;466:128–32. 10.1038/nature09143. 10.1038/nature09143 PMC2967763 20596025

[70] Riou-Khamlichi C. Cytokinin Activation of Arabidopsis Cell Division Through a D-Type Cyclin. Science (80-). 1999;283:1541–4. 10.1126/science.283.5407.1541. 10.1126/science.283.5407.1541 10066178

[71] Dewitte W, Scofield S, Alcasabas AA, Maughan SC, Menges M, Braun N, et al. Arabidopsis CYCD3 D-type cyclins link cell proliferation and endocycles and are rate-limiting for cytokinin responses. Proc Natl Acad Sci. 2007;104:14537–42. 10.1073/pnas.0704166104. 10.1073/pnas.0704166104 PMC1964848 17726100

[72] Dello Ioio R, Nakamura K, Moubayidin L, Perilli S, Taniguchi M, Morita MT, et al. A genetic framework for the control of cell division and differentiation in the root meristem. Science. 2008;322:1380–4. 10.1126/science.1164147. 10.1126/science.1164147 19039136

[73] Moubayidin L, Di Mambro R, Sabatini S. Cytokinin–auxin crosstalk. Trends Plant Sci. 2009;14:557–62. 10.1016/j.tplants.2009.06.010. 10.1016/j.tplants.2009.06.010 19734082

[74] Tanimoto E. Regulation of Root Growth by Plant Hormones—Roles for Auxin and Gibberellin. CRC Crit Rev Plant Sci. 2005;24:249–65. 10.1080/07352680500196108.

[75] Almada R, Cabrera N, Casaretto JA, Ruiz-Lara S, González Villanueva E. VvCO and VvCOL1, two CONSTANS homologous genes, are regulated during flower induction and dormancy in grapevine buds. Plant Cell Rep. 2009;28:1193–203. 10.1007/s00299-009-0720-4. 10.1007/s00299-009-0720-4 19495771

[76] Srinivasan C, Mullins MG. Effects of Temperature and Growth Regulators on Formation of Anlagen, Tendrils and Inflorescences in Vitis vinifera L. Ann Bot. 1980;45:439–46. 10.1093/oxfordjournals.aob.a085842.

[77] Arana M V., Marin-de la Rosa N, Maloof JN, Blazquez MA, Alabadi D. Circadian oscillation of gibberellin signaling in Arabidopsis. Proc Natl Acad Sci. 2011;108:9292–7. 10.1073/pnas.1101050108. 10.1073/pnas.1101050108 PMC3107313 21576475

[78] Boss PK, Buckeridge EJ, Poole A, Thomas MR. New insights into grapevine flowering. Funct Plant Biol. 2003;30:593. 10.1071/FP02112. 10.1071/FP02112 32689045

[79] Díaz-Riquelme J, Grimplet J, Martínez-Zapater JM, Carmona MJ. Transcriptome variation along bud development in grapevine (Vitis vinifera L.). BMC Plant Biol. 2012;12:181. 10.1186/1471-2229-12-181. 10.1186/1471-2229-12-181 PMC3519583 23035802

[80] Martin RC, Asahina M, Liu PP, Kristof JR, Coppersmith JL, Pluskota WE, et al. The regulation of post-germinative transition from the cotyledon- to vegetative-leaf stages by microRNA-targeted squamosa promoter-binding protein like13 in Arabidopsis. Seed Sci Res. 2010;20:89–96. 10.1017/S0960258510000073.

[81] Wang S, Wu K, Yuan Q, Liu X, Liu Z, Lin X, et al. Control of grain size, shape and quality by OsSPL16 in rice. Nat Genet. 2012;44:950–4. 10.1038/ng.2327. 10.1038/ng.2327 22729225

[82] Wang H, Nussbaum-Wagler T, Li B, Zhao Q, Vigouroux Y, Faller M, et al. The origin of the naked grains of maize. Nature. 2005;436(7051):714–9. 10.1038/nature03863. 10.1038/nature03863 PMC1464477 16079849

[83] Hartmann U, Hohmann S, Nettesheim K, Wisman E, Saedler H, Huijser P. Molecular cloning of SVP: a negative regulator of the floral transition in Arabidopsis. Plant J. 2000;21:351–60. 10.1046/j.1365-313x.2000.00682.x. 10.1046/j.1365-313x.2000.00682.x 10758486

[84] Diaz-Riquelme J, Lijavetzky D, Martinez-Zapater JM, Carmona MJ. Genome-Wide Analysis of MIKCC-Type MADS Box Genes in Grapevine. Plant Physiol. 2009;149:354–69. 10.1104/pp.108.131052. 10.1104/pp.108.131052 PMC2613716 18997115

[85] Turnbull C. Long-distance regulation of flowering time. J Exp Bot. 2011;62:4399–413. 10.1093/jxb/err191. 10.1093/jxb/err191 21778182

[86] Sudhakar P, Latha P, Reddy PV. Plant pigments. In: Phenotyping Crop Plants for Physiological and Biochemical Traits. Elsevier; 2016. p. 121–7. 10.1016/B978-0-12-804073-7.00015-6.

[87] Yang Y, Mao L, Jittayasothorn Y, Kang Y, Jiao C, Fei Z, et al. Messenger RNA exchange between scions and rootstocks in grafted grapevines. BMC Plant Biol. 2015;15:1–14. 10.1186/s12870-015-0626-y. 10.1186/s12870-015-0626-y PMC4612405 26480945

[88] Anders S, Pyl PT, Huber W. HTSeq--a Python framework to work with high-throughput sequencing data. Bioinformatics. 2015;31:166–9. 10.1093/bioinformatics/btu638. 10.1093/bioinformatics/btu638 PMC4287950 25260700

[89] Robinson MD, McCarthy DJ, Smyth GK. edgeR: a Bioconductor package for differential expression analysis of digital gene expression data. Bioinformatics. 2010;26:139–40. 10.1093/bioinformatics/btp616. 10.1093/bioinformatics/btp616 PMC2796818 19910308

[90] Joung J-G, Corbett AM, Fellman SM, Tieman DM, Klee HJ, Giovannoni JJ, et al. Plant MetGenMAP: An Integrative Analysis System for Plant Systems Biology. Plant Physiol. 2009;151:1758–68. 10.1104/pp.109.145169. 10.1104/pp.109.145169 PMC2786002 19819981

[91] Supek F, Bošnjak M, Škunca N, Šmuc T. REVIGO Summarizes and Visualizes Long Lists of Gene Ontology Terms. PLoS One. 2011;6:1–9. 10.1371/journal.pone.0021800. 10.1371/journal.pone.0021800 PMC3138752 21789182

[92] Berardini TZ, Reiser L, Li D, Mezheritsky Y, Muller R, Strait E, et al. The arabidopsis information resource: Making and mining the “gold standard” annotated reference plant genome. genesis. 2015;53:474–85. 10.1002/dvg.22877. 10.1002/dvg.22877 PMC4545719 26201819

[93] Goodstein DM, Shu S, Howson R, Neupane R, Hayes RD, Fazo J, et al. Phytozome: a comparative platform for green plant genomics. Nucleic Acids Res. 2012;40:D1178–86. 10.1093/nar/gkr944. 10.1093/nar/gkr944 PMC3245001 22110026

